# Silibinin Upregulates CXCR4 Expression in Cultured Bone Marrow Cells (BMCs) Especially in Pulmonary Arterial Hypertension Rat Model

**DOI:** 10.3390/cells9051276

**Published:** 2020-05-21

**Authors:** Tingting Zhang, Nanako Kawaguchi, Kunikazu Tsuji, Emiko Hayama, Yoshiyuki Furutani, Hisashi Sugiyama, Toshio Nakanishi

**Affiliations:** 1 Pediatric Cardiology and Adult Congenital Cardiology, Tokyo Women’s Medical University, Tokyo 162-8666, Japan; 13572273657@163.com (T.Z.); emiko-ha@twmu.ac.jp (E.H.); yfurutani@twmu.ac.jp (Y.F.); sugiyama.hisashi@twmu.ac.jp (H.S.); nakanishi.toshio@twmu.ac.jp (T.N.); 2 Department of Cartilage Regeneration, Tokyo Medical and Dental University, Tokyo 113-8510, Japan; tsuji.orj@tmd.ac.jp

**Keywords:** pulmonary arterial hypertension, silibinin, CXCR4, bone marrow, granulocyte, monocyte-macrophage

## Abstract

Previously we reported that silibinin ameliorated pulmonary arterial hypertension (PAH) in rat PAH models, possibly through the suppression of the CXCR4/SDF-1, until the point where PAH became a severe and irreversible condition. To further investigate how silibinin ameliorates PAH, we first attempted to clarify its effect on bone marrow cells (BMCs), since the CXCR4/SDF-1 axis is known to regulate stem cell migration and attachment in BM niches. Rat PAH models were established through a combination of a single subcutaneous injection of monocrotaline (MCT) and chronic hypoxic conditions (10% O_2_). BMCs were harvested and cultured, and reverse transcription-quantitative polymerase chain reaction (RT-qPCR) and flow cytometry (FCM) were performed to investigate whether silibinin affected CXCR4 expression. Silibinin upregulated the gene expression of stem cell related markers CXCR4, SDF-1, SCF, and c-Kit, inflammatory markers IL-6 and TNFα, mesenchymal stem cell (MSC)-related markers CD44 and CD29, and the granulocyte/monocyte-macrophage marker CD14 in cultured BM in PAH rats, but not in normal rats, except CXCR4. FCM showed that silibinin increased the CXCR4-positive cell population in a granulocyte fraction of cultured BMCs. However, immunohistochemical (IHC) staining showed no significant change in CXCR4 expression in the BM of the tibias. These results suggest that silibinin increases the expression of CXCR4 in BM, and the increased CXCR4-positive cells could be granulocytes/monocyte-macrophages.

## 1. Introduction

Pulmonary arterial hypertension (PAH) is a severe and fatal clinical syndrome characterized by high blood pressure and vascular remodeling in the pulmonary arterioles. Right ventricular overload, right heart failure, and death may result from severe pathologies [1,2,3]. No effective treatment to cure the disease has yet been discovered. Recent studies of PAH treatment have focused on gene and cell therapy in animal models, as these treatments are considered to attenuate pulmonary vascular remodeling [4,5]. The main classes of drugs which have been widely used to treat PAH include endothelin-1 receptor antagonists, phosphodiesterase type 5 inhibitors, and prostacyclins [6]. For more treatment options, we focused on C-X-C chemokine receptor type 4 (CXCR4) and performed pharmacological analysis of a CXCR4 inhibitor, silibinin, to treat PAH.

CXCR4 is expressed in stem/progenitor cells, including endothelial and smooth muscle progenitors [7,8]. The ligand of CXCR4 is stromal cell-derived factor-1 (SDF-1), also called C-X-C chemokine ligand 12 (CXCL12). CXCR4-positive cells have recently been implicated in the development of pulmonary arterial hypertension (PAH) [8,9,10,11,12,13]. We previously reported that CXCR4 expression is significantly higher in the pulmonary arteries of PAH rats treated with monocrotaline (MCT) and hypoxia than in normal rats [14]. Therefore, we verified that a CXCR4 inhibitor, silibinin, can ameliorate PAH, possibly through the suppression of the CXCR4/SDF-1 axis, until the point where PAH becomes a severe and irreversible condition [15]. Furthermore, the small molecule CXCR4 inhibitor AMD3100 (Plerixafor) has been reported to attenuate the development of PAH [9,12,13]. Silibinin is derived from the seeds of the milk thistle plant *Silybum marianum* L. [16,17]. It is usually used to treat liver diseases [18,19,20], and has been reported to have antineoplastic potential [21,22,23]. Silibinin is likely to affect the stem cells in bone marrow (BM), since the CXCR4/SDF-1 axis is known to be involved in stem cell homing in BM [7,8]. Previous reports suggest that BM cells contribute to the development of pathogenesis of PAH using GFP-labeled BM transplantation in both genetic models [24] and hypoxia-induced models [25]. However, there are no reports that these BM cells are related to CXCR4. Long term low-dosage Plerixafor affects BM cell constitution in WHIM syndrome, which is caused by a CXCR4 mutation [26]. In the present study, we therefore investigated the effect of silibinin on the BM cells of normal rats and PAH rat models.

## 2. Materials and Methods

### 2.1. Animal Preparation

All PAH models were established as described previously [14,27], by subcutaneously injecting rats with a single dose of MCT (Sigma-Aldrich, St. Louis, MO, USA) and maintaining them in a hypoxic chamber (10% O_2_) (Everest Summit II Altitude Generator: Hypoxico Inc., New York, NY, USA) for two weeks, using male, 7–8-week-old Sprague–Dawley rats weighing 180–250 g (Tokyo Experimental Animal Company, Tokyo, Japan). MCT was dissolved in 1 N HCl, neutralized with 1 N NaOH, and diluted with distilled water to 20 mg/mL. A dose of 60 mg/kg (3 mL/kg) body weight was administered to the rats. All rats had unlimited access to food and water and were weighed weekly. Silibinin was suspended in 0.5% carboxymethyl cellulose (CMC) sodium salt water (Wako Pure Chemical Industries, Ltd., Tokyo, Japan) for oral dosage. For in vivo experiments, 16 rats were randomly assigned to a normal-control group (*n* = 4), normal-silibinin group (*n* = 4), PAH-control group (*n* = 4), and PAH-silibinin group (*n* = 4). CMC water was dosed once per day for the rats in the normal-control group and PAH-control group, and silibinin (Sigma-Aldrich, 200 mg/kg) with CMC water was dosed once per day for the rats in the normal-silibinin group and PAH-silibinin group. All rats were sacrificed under isoflurane inhalation (2.0% mixed with air, at an inhalation rate of approximately 350 mL/min) after the experiments were completed. All animal experiment protocols were approved by the Institutional Animal Experiment Committee of the Tokyo Women’s Medical University (AE18-111, April 5, 2018, AE19-031, March 15, 2019). All animal procedures were in accordance with the ethical standards of the institution and conformed to the guidelines from Directive 2010/63/EU of the European Parliament on the protection of animals used for scientific purposes or the current NIH guidelines (NIH publication No. 85–23).

### 2.2. Bone Marrow Cell (BMC) Preparation

Bone marrow cells (BMCs) were flushed out from the tibias, collected, and cultured on 6-well plates in MEM medium (Sigma-Aldrich) supplemented with 10% fetal bovine serum (BD Biosciences Clontech, Palo Alto, CA, USA), 100 μg/mL streptomycin, and 100 units/mL penicillin (Sigma-Aldrich). All cells were cultured at 37 °C in a humidified CO_2_ incubator.

For in vitro analysis, the cultured BMCs from PAH rats were divided equally into control (*n* = 7 wells), silibinin (*n* = 5 wells), and AMD3100 (*n* = 4 wells) treatments. After two days, the medium was changed and dimethyl sulfoxide (DMSO) (Sigma-Aldrich), 5 μM silibinin dissolved in DMSO, or 5 μM AMD3100 (Abcam, Cambridge, UK) was added to each well. One day later, the cells were collected and reverse transcription-quantitative polymerase chain reaction (RT-qPCR) was performed.

To investigate the effect of temporal differences of silibinin treatment, 2 normal rats and 2 PAH rats were sacrificed for the in vitro experiment. The BMCs of each rat were harvested and divided in two. The first half of the BMCs were cultured, with two dishes from each rat. After two days, the medium was changed and DMSO (control) or 5 μM silibinin were added to each dish, creating four treatments: normal-control, PAH-control, normal-silibinin, and PAH-silibinin. One day later, the cells were collected and flow cytometry (FCM) was performed.

The other half of the BMCs were divided into six treatments: short-term normal, short-term PAH, long-term normal 1, long-term PAH 1, long-term normal 2, and long-term PAH 2. For short-term and long-term 1 treatments, cells were cultured for two days, after which the medium was changed and DMSO (control) or silibinin was added; for short-term treatments, cells were collected one day later for RT-qPCR; for long-term 1 treatment, cells were collected five days later for RT-qPCR. For long-term 2 treatment, cells were cultured for six days, after which the medium was changed and DMSO (control) or silibinin was added; cells were collected for RT-qPCR one day later. The details of BMC preparation are presented in graphical representation in Figure S1.

### 2.3. Reverse Transcription-Quantitative Polymerase Chain Reaction

Reverse transcription-quantitative polymerase chain reaction (RT-qPCR) was performed as previously described [14]. β-actin mRNA expression was measured for normalization. mRNA expression was normalized to β-actin expression using the 2*^−∆∆CT^* equation. Primer sequences are listed in Table S1.

### 2.4. FCM

Cultured BMCs were harvested and incubated in 0.5% bovine serum albumin (BSA; Seikagaku Kogyo, Tokyo, Japan) for 1 h, then stained with the CXCR4 (1:1000; Abcam) primary antibody for 1 h and rat anti-IgG1-FITC (eBioscience, San Diego, CA, USA) for 30 min at room temperature, following fixation. The equivalent negative control procedure, and one using the secondary antibody only, were performed at the same time. A total of 1  ×  10^4^ events were examined for each subject. The samples were measured and analyzed by Moflow (Beckman Coulter, Brea, CA, USA) or FACS Verse and BD Bioscience FACSuite^TM^ (Frankin Lake, NJ, USA).

### 2.5. Immunohistochemical Analysis

Following the in vivo experiments, all rats were sacrificed, and the tibias were harvested and prefixed in 4% paraformaldehyde for 1 week. They were then stored in 70% ethanol for 1 week and incubated with a decalcifying solution of 10% ethylene diamine tetra-acetic acid (EDTA, pH = 7.4) for 28 days. Decalcification was performed at 4 °C and the decalcifying solutions were changed every 2 days. 

Immunohistochemical staining was performed as previously described. Primary antibodies against CXCR4 (1:1000; Abcam) were used. ImageJ (National Institutes of Health, Bethesda, MD, USA) was used to calculate the percentage of CXCR4 positive cells. We randomly chose 15 microscopic areas per section from each rat.

### 2.6. Hemodyamic Analysis

Right ventricular systolic pressure (RVSP) and Fulton index (weight ratio of RV to LV + S) were measured at the end of the two weeks of experiments as previously described [14,28].

### 2.7. Statistical Analyses

Quantitative data are expressed as mean ± standard deviation. Differences between two groups were analyzed by t-test. Comparison of time-course was analyzed by one-way analysis of variance (ANOVA) and Bonferroni’s post hoc test. SPSS software (SPSS, Inc., Chicago, IL, USA) was used for all statistical analyses. A *p* value < 0.05 was considered statistically significant. 

## 3. Results

### 3.1. Effect of Silibinin in the BMCs of PAH Models in Gene Expression Level

**Figure 1:**
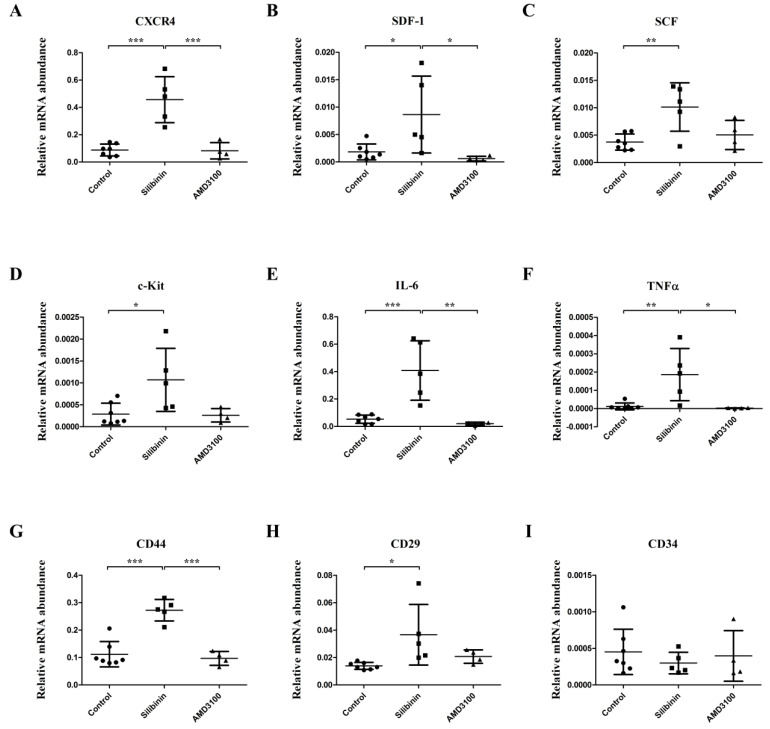
Comparison of gene expression levels of stem cell-related markers and inflammatory markers among control, silibinin, and AMD3100 treatment cultures from pulmonary arterial hypertension (PAH) rat bone marrow cells (BMCs). Silibinin significantly upregulated the expression of CXCR4 (**A**), SDF-1 (**B**), SCF (**C**), c-Kit (**D**), inflammatory markers IL-6 (**E**), TNFα (**F**), MSC-related markers CD44 (**G**), and CD29 (**H**). However, significant differences were not observed between the control group and AMD3100 group. Silibinin and AMD3100 did not upregulate the expression of HSC-related marker CD34 (I). * *p* < 0.05, ** *p* < 0.01, *** *p* < 0.001. Treatment groups consisted of seven samples in the control culture, five samples in the silibinin culture, and four samples in the AMD3100 culture.

All rats survived and remained active during the experiment. RT-qPCR revealed that silibinin upregulated the gene expression of: stem cell markers CXCR4 (Figure 1A, *p* < 0.001), SDF-1 (Figure 1B, *p* < 0.05), SCF (Figure 1C, *p* < 0.01), and c-Kit (Figure 1D, *p* < 0.05); inflammatory markers IL-6 (Figure 1E, *p* < 0.001) and TNFα (Figure 1F, *p* < 0.01); mesenchymal stem cell (MSC)-related markers CD44 (Figure 1G, *p* < 0.001) and CD29 (Figure 1H, *p* < 0.05) in BM. However, no significant difference was found between the control group and the AMD3100 group (Figure 1A–H) as well as for the level of hematopoietic stem cell (HSC)-related marker CD34 between the three groups (Figure 1I). Notably, significant differences between the silibinin group and the AMD3100 group were found in the gene expression of CXCR4 (Figure 1A, *p* < 0.001), SDF-1 (Figure 1B, *p* < 0.05), IL-6 (Figure 1E, *p* < 0.01), TNFα (Figure 1F, *p* < 0.05), and CD44 (Figure 1G, *p* < 0.001). RT-qPCR revealed that silibinin could only significantly upregulate the expression of CXCR4 in the BM of normal rats in stem cell-related markers (CXCR4, SDF-1, SCF, c-Kit) and inflammatory markers (IL-6, TNFα). The details are shown in Figure S2.

**Figure 2:**
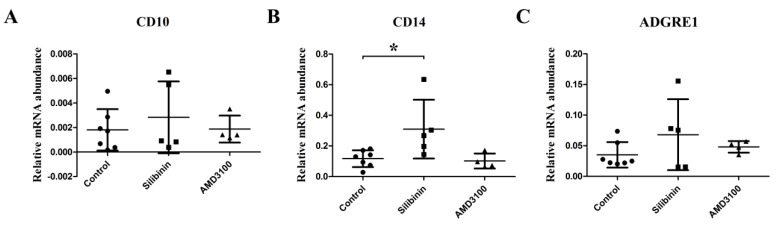
Comparison of gene expression levels of granulocyte and monocyte-macrophage cell markers among control, silibinin, and AMD3100 treatment cultures. (**A**) Silibinin and AMD3100 did not upregulate the expression of lymphocyte marker-CD10. (**B**) Silibinin significantly upregulated the expression of monocyte-macrophage and activated granulocyte marker-CD14 in BM. However, no significant difference was found between the control culture and the AMD3100 culture. (**C**) Silibinin and AMD3100 did not upregulate the expression of monocyte-macrophage cell marker-ADGRE1. * *p* < 0.05. Treatment groups consisted of seven samples in the control culture, five samples in the silibinin culture, and four samples in the AMD3100 culture.

Silibinin upregulated the expression of granulocyte cell and monocyte-macrophage cell marker-CD14, but significant differences were not observed in mature neutrophil marker-CD10 and monocyte-macrophage cell marker-adhesion G protein-coupled receptor E1 (ADGRE1) (Figure 2A–C).

### 3.2. CXCR4-Positive Cell Population Association with Granulocytes/Monocyte-Macrophage

**Figure 3:**
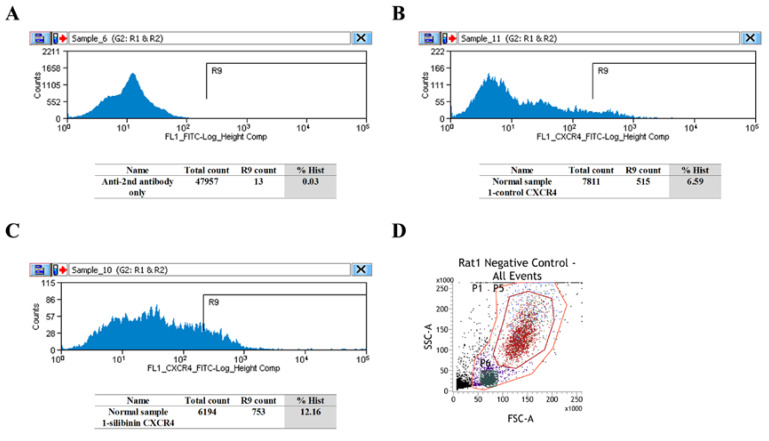

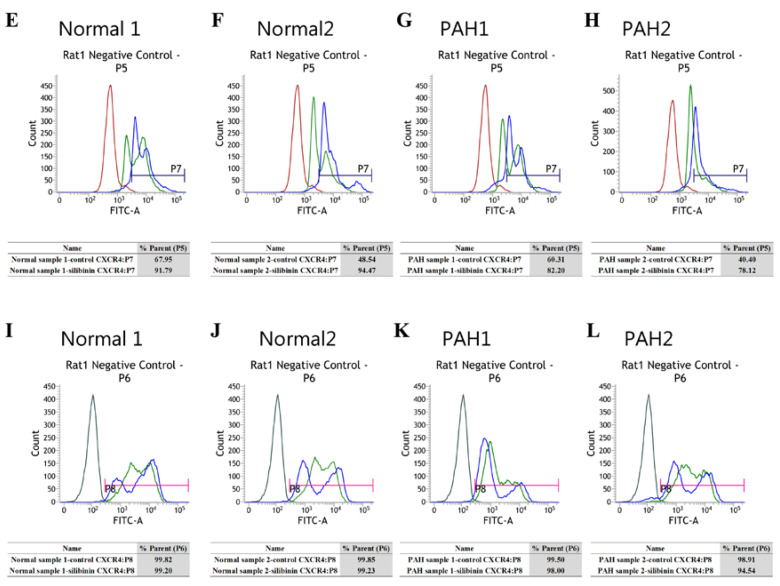
Flow cytometry (FCM) evaluation of CXCR4 in normal and PAH rats. (**A**–**C**) In the BM of normal rats, silibinin upregulated the percentage of CXCR4-positive cells. Secondary antibody only (**A**), normal 1-control (**B**), and normal 1-silibinin (**C**). (**D**) Negative control of granulocytes (P5 area, red) and negative control of monocyte-macrophages (P6 area, dark green). (**E**–**H**) In the BM of normal and PAH rats, silibinin increased the percentage of CXCR4-positive cells in the granulocyte fraction. Negative control (red), normal 1-control (light green), and normal 1-silibinin (blue) (**E**). Negative control (red), normal 2-control (light green), and normal 2-silibinin (blue) (**F**). Negative control (red), PAH 1-control (light green), and PAH 1-silibinin (blue) (**G**). Negative control (red), PAH 2-control (light green), and PAH 2-silibinin (blue) (**H**). (**I**–**L**) In the BM of normal and PAH rats, the percentage of CXCR4-positive cells was not altered in the monocyte-macrophage fraction. Negative control (dark green), normal 1-control (light green), and normal 1-silibinin (blue) (**I**). Negative control (dark green), normal 2-control (light green), and normal 2-silibinin (blue) (**J**). Negative control (dark green), PAH 1-control (light green), and PAH 1-silibinin (blue) (**K**). Negative control (dark green), PAH 2-control (light green), and PAH 2-silibinin (blue) (**L**). Treatment groups consisted of two samples in each culture.

FCM results demonstrated that in the BM of normal rats, silibinin upregulated the cell population percentage of CXCR4-positive cells (Figure 3A–C and Figure S3). In both normal and PAH rats, increased CXCR4-positive cells were observed in the granulocyte fraction (P5 in Figure 3D–H), and almost all cells were CXCR4-positive in the monocyte-macrophage fraction (P6 in Figure 3D,I–L).

To observe temporal changes in the upregulation of CXCR4 in BMCs, BMCs were divided into three cultures to perform RT-qPCR as described in the Materials and Methods. We divided them into short-term (4 day) and long-term (8 day) cultures because granulocytes would not survive in the long-term cultured BMCs. The difference between PAH long-term groups 1 and 2 was the point in time at which silibinin was added to the dishes.

**Figure 4:**
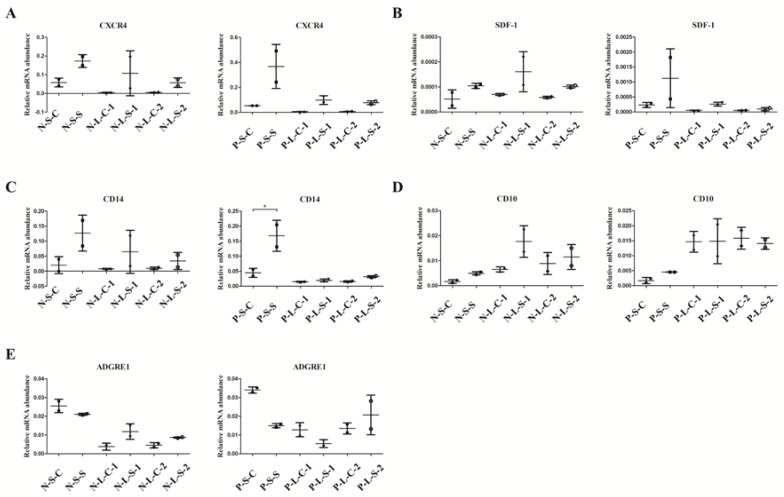
Gene expression in vitro demonstrating the positive relationship between CD14 and CXCR4. (**A**-**C**) The gene expression of CXCR4 (**A**) and SDF-1 (**B**) showed similar trends to that of CD14 (**C**). Short-term cultured BMCs showed increased CD14 expression in PAH rats, while long-term cultured BMCs did not (**C**). (**D**,**E**) The gene expression of CD10 (**D**) and ADGRE1 (**E**) did not show similar trends to those of CXCR4 (**A**) and SDF-1 (**B**). N-S-C: normal-short-control culture, N-S-S: normal-short-silibinin culture, N-L-C-1: normal-long-control 1 culture, N-L-S-1: normal-long-silibinin-1 culture, N-L-C-2: normal-long-control 2 culture, N-L-S-2: normal-long-silibinin-2 culture, P-S-C: PAH-short-control culture, P-S-S: PAH-short-silibinin culture, P-L-C-1: PAH-long-control 1 culture, P-L-S-1: PAH-long-silibinin-1 culture, P-L-C-2: PAH-long-control 2 culture, P-L-S-2: PAH-long-silibinin-2 culture, * *p* < 0.05. Treatment cultures consisted of two samples in each group.

RT-qPCR results also demonstrated that for both normal and PAH rats, the gene expression of CXCR4 and SDF-1 (Figure 4A,B) in cultured BMCs showed similar trends to that of CD14 (Figure 4C), rather than that of CD10 and ADGRE1 (Figure 4D,E). Furthermore, CD14 gene expression in short-term cultured BMCs increased (Figure 4C, *p* < 0.05) in PAH rats; however, there was no increase observed in long-term cultured BMCs. These results suggest that CXCR4 may come from CD14-positive cells.

### 3.3. Immunohistochemical and Hemodynamic Studies

**Figure 5:**
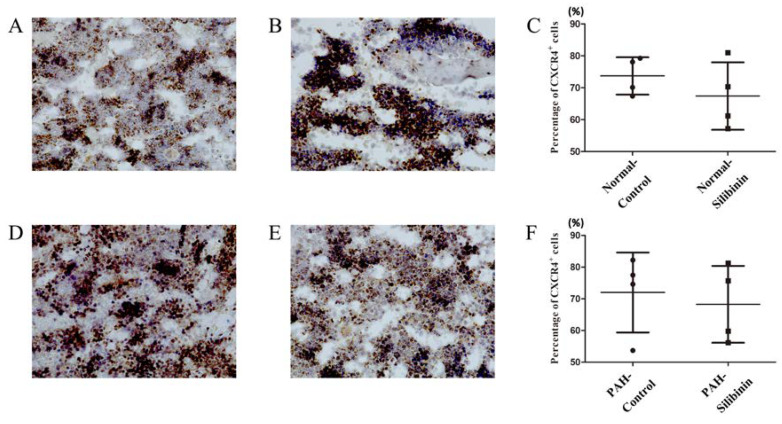
Immunohistochemical evaluation of CXCR4 in BM of normal and PAH rats. (**A**,**B**) CXCR4-positive cells in normal rats without (**A**) or with (**B**) silibinin. (**C**) No significant difference was observed in these groups. (**D**,**E**) CXCR4-positive cells were observed in PAH rats both without (**D**) and with (**E**) silibinin. (**F**) No significant difference was observed in these groups. Each group consisted of four rats.

Immunohistochemical analysis showed no increase in CXCR4-positive cells caused by silibinin treatment in either normal (Figure 5A–C) or PAH rats (Figure 5D–F).

**Figure 6:**
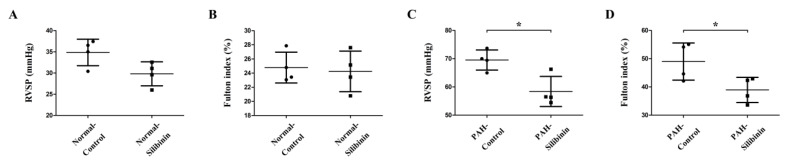
Hemodynamic studies of PAH without or with silibinin treatment. (**A**,**B**) Silibinin did not decrease RVSP (**A**) and the Fulton index (**B**) after two weeks of treatment in the normal-silibinin group compared to the normal-control group. (**C**,**D**) Silibinin significantly decreased RVSP (**C**) and the Fulton index (**D**) after two weeks of treatment in the PAH-silibinin group compared to the PAH-control group. * *p* < 0.05. Each group consisted of four rats.

In the silibinin oral dosage experiment, no significant difference was observed between the normal-silibinin group and the normal-control group (Figure 6A,B). Silibinin significantly decreased RVSP (Figure 6C, *p* < 0.05) and the Fulton index (Figure 6D, *p* < 0.05) after two weeks of treatment in the PAH-silibinin group compared with the PAH-control group.

## 4. Discussion

There were six main findings in the present study. First, silibinin upregulated the expression of some stem cell related markers such as CXCR4, MSC cell related markers, and inflammation markers in the BMCs of PAH rats. In contrast with these results, no significant difference was observed in the HSC-related marker. FCM results also demonstrated that silibinin led to an increase in the percentage of CXCR4-positive cells in the BM of both normal and PAH rats.

Second, silibinin upregulated the gene expression of CD14 in PAH rats, which is a marker of the monocyte-macrophage lineage and activated granulocytes [29]. The gene expression of CXCR4 and SDF-1 showed similar trends to the gene expression of CD14 following silibinin treatment in both normal and PAH rats. FCM results showed an increased percentage of CXCR4-positive cells in the granulocyte fraction but not the monocyte-macrophage fraction following silibinin treatment. The gene expression of CD14 was increased in short-term cultured BMCs, but not in long-term cultured BMCs. These results suggest that the increase in CXCR4-positive cells may come from CD14-positive cells.

Third, silibinin upregulated the expression of inflammation markers in cultured BMCs.

Fourth, CXCR4 inhibitors, AMD3100 and silibinin, modulated gene expression to different levels in cultured BMCs.

Fifth, immunohistochemical analysis showed that silibinin did not enhance the expression of CXCR4-positive cells in the BM of either normal or PAH rats.

Finally, silibinin treatment led to a decrease in RVSP and Fulton index in PAH rats, as we previously reported [15], but had no effect in normal rats.

It has previously been reported that the CXCR4/SDF-1 axis plays an essential role in retaining stem cells in the BM [30,31]. Thus, a CXCR4 inhibitor may induce stem cell mobilization from the BM into the blood circulation, causing the number of stem cells in the BM to decrease. In the present study, upregulation of stem cell related markers CXCR4, SDF-1, SCF, and c-Kit, and MSC cell related markers CD44 and CD29 in the BM of PAH rats was observed. FCM demonstrated similar results for CXCR4-positive cells. A plausible explanation is that, in our study, silibinin was added into cell dishes; therefore, CXCR4-positive cells remained in cell dishes but did not migrate from the BM as in vivo. Indeed, in the in vivo experiments, CXCR4-positive cells did not increase in the BM, which might indicate that CXCR4-positive cells migrate to the peripheral tissues. AMD3100 revealed hematopoietic stem cell migration via the SDF-1/CXCR4 axis [32]. Furthermore, the observed upregulation of inflammation marker gene levels suggests that silibinin can induce inflammatory stimulation in the BM of PAH rats. These inflammatory cells may migrate to damaged tissues in vivo. Taken together, these results show that silibinin increased the numbers of CXCR4-positive stem cells, granulocytes, and monocyte-macrophages in the BM and migrated to the tissues damaged by hypoxia. It is still not clear whether CXCR4-positive cells rescue the damaged tissues or inhibit the rescue by inducing inflammation. Our previous results showed that expression of CXCR4 and inflammatory markers was decreased in the PAH rodent model of the lungs through silibinin treatment [15]. We think that the roles of CXCR4 and inflammatory cells are complicated. These cells may rescue the damaged tissues but may also contribute to the development of PAH. If we can compare the effect of silibinin administrated orally against that administrated nasally, directly targeting to the lungs, it may be more clear.

CD14 is known as a marker of the monocyte-macrophage lineage and activated granulocytes [29]; CD10 is known as an early lymphocyte marker in mice [33]; ADGRE1 is known as a monocyte-macrophage cell marker [34]. In the present study, the gene expression of CD14 was upregulated following silibinin treatment, and showed a similar trend to the gene expression of CXCR4 and SDF-1. FCM results showed an increased percentage of CXCR4-positive cells in the granulocyte fraction. Furthermore, the RT-qPCR results showed that CXCR4 expression was higher in short-term cultured cells but not in the long-term cultured BMCs, suggesting that the CXCR4-positive cells may be granulocytes. No significant difference was observed in the expression of ADGRE1 and CD10. However, in the macrophage-fractioned cell population, almost 100% of the cells were CXCR4- positive without silibinin treatment. Therefore, we cannot exclude the possibility that we could not detect the increase in CXCR4-positive cells in this fraction. We concluded that the increased CXCR4-positive cells were likely to be CD14-positive cells, which were at least activated granulocytes and possibly were monocyte-macrophages. These increased CXCR4-positive cells may be involved in inflammation. The gene expression of CD14, ADGRE, CXCR4, and SDF-1 declined in the long-term cultured BMCs, but not in the short-term cultured BMCs, while the gene expression of CD10 increased in the long-term cultured BMCs as well as in short-term cultured BMCs. One possible explanation is that CD14, ADGRE, CXCR4, and SDF-1-expressing cells, which might be monocyte-macrophage and activated granulocytes, could not survive in the long-term BMC cultures, so they decreased over time; however, CD10-positive cells were different, increasing over time, and these may have grown to be mature lymphocytes. CD44 is also a cell surface receptor for hyaluronic acid and is involved in monocyte differentiation. Taken together, our results may indicate that silibinin increases CXCR4-positive cells and develops monocytes and granulocytes in BM.

The anti-inflammatory effect of silibinin has been reported in different kinds of disease and models [15,35,36]. In the present study, upregulation of inflammation markers IL-6 and TNFα in the BM of PAH rats was observed. These results suggest that silibinin has an anti-inflammatory effect in the PAH pulmonary artery (PA), but increases the number of inflammation cells in the BM. Like CXCR4-positive cells, these cells may rescue the damaged tissues but may also contribute to damage progression.

CXCR4 inhibitors, AMD3100 and silibinin, modulated gene expression at different levels in our study. Notably, there was a significant difference in the increase of CXCR4 expression between silibinin and AMD3100, suggesting a more potent effect with silibinin than with AMD3100 [37]. Previously, we speculated that silibinin and AMD3100 are different CXCR4 inhibitors, with different structures and mechanisms of binding, leading to different efficacies in PAH treatment. It was reported that AMD3100 specifically blocks SDF-1 but not phorbol ester-induced internalization of CXCR4 [38]; thus, one possible reason is that silibinin may inhibit CXCR4 in a different manner to AMD3100 in in vitro experiments. On the other hand, silibinin can have other efficiencies to induce inflammation signal transduction in BM.

In immunohistochemical experiments we did not observe any effect of silibinin on the expression of CXCR4-positive cells. One possible reason is that the CXCR4-positive cells aggregated, and therefore we could not count them accurately. However, further studies are necessary.

Silibinin treatment led to a decrease in RVSP and Fulton index in PAH rats, but not in normal rats, which suggests that silibinin could ameliorate PAH models only, and leave normal models unaffected.

The present study has some limitations. First, we observed CXCR4 expression following silibinin treatment in the BM only; however, the mechanism of CXCR4 inhibition in BM remains unclear. Second, the expression of CXCR4 in vivo may be different to that used in the in vitro experiments, which requires further studies to investigate. Last, side effects of silibinin in PAH patients are still unknown. The CD inflammatory markers are expressed differently between humans and mice (or other rodents). Therefore, further studies are necessary and may lead to the direction for more options for PAH treatment. At the same time, whether the treatment with silibinin induces any change in the circulating cells in vivo needs to be discussed, since these circulating cells may be possible progenitors of lung inflammatory cells.

## Supplementary Materials

The following are available online at https://www.mdpi.com/2073-4409/9/5/1276/s1, Figure S1: Graphical representation of BMC preparation. Figure S2: Comparison of gene expression levels of stem cell related markers and inflammatory markers between control and silibinin treatment groups of normal samples, Figure S3: FCM evaluation of CXCR4 in normal rats using Moflo. Table S1. Primers used for real-time PCR.
