# Supplementary material for: Silibinin Upregulates CXCR4 Expression in Cultured Bone Marrow Cells (BMCs) Especially in Pulmonary Arterial Hypertension Rat Model
Source: Cells. 2020 May 21;9(5):1276. doi: 10.3390/cells9051276 (PMC7290890; doi:10.3390/cells9051276)
Supplement: Supplementary file 1 [file cells-09-01276-s001.pdf]

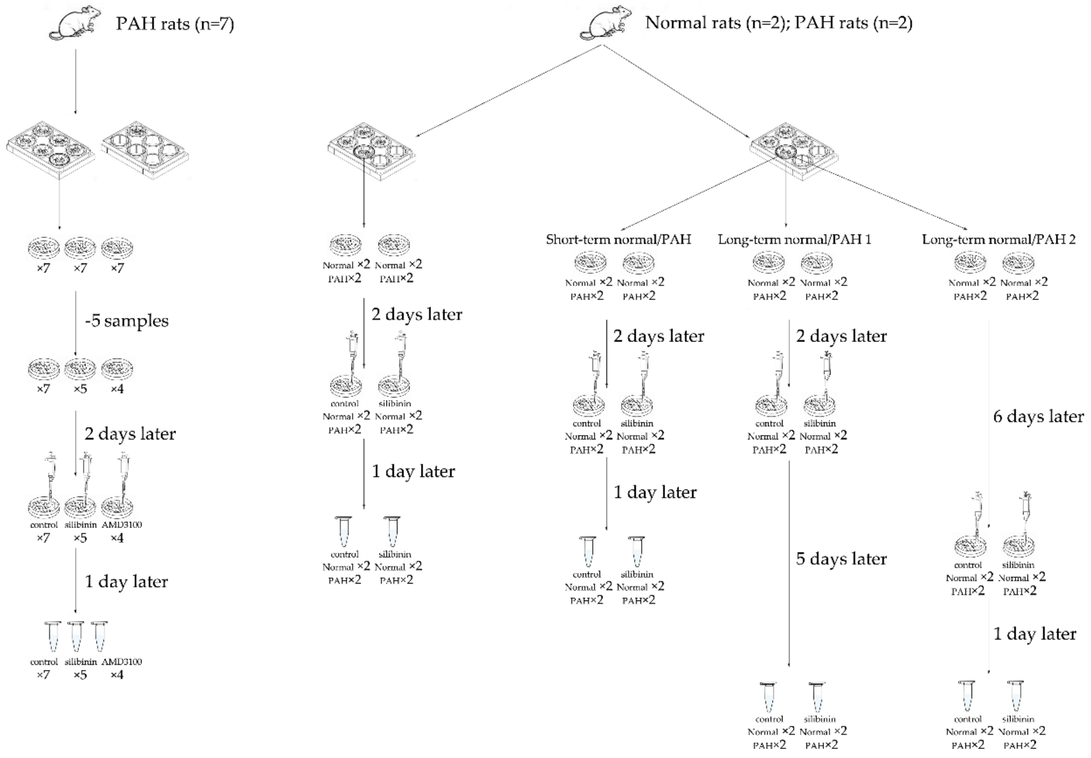

Figure S1. Graphical representation of BMCs preparation.

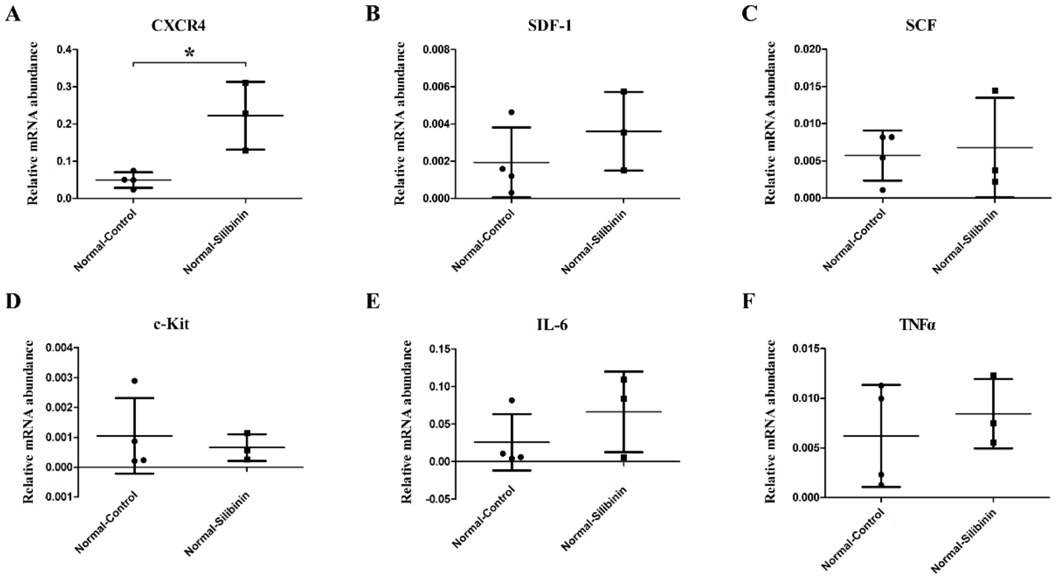

Figure S2. Comparison of gene expression levels of stem cell-related markers and inflammatory markers between control and silibinin treatment groups of normal samples. Silibinin significantly upregulated the expression of CXCR4 (**A**) in BM. However, silibinin did not upregulate the expression of stem cell markers-SDF-1 (**B**), SCF (**C**), c-Kit (**D**), inflammatory markers IL-6 (**E**) and TNFα (**F**) in BM. *$p < 0.05$. Treatment groups consisted of four samples in Normal-control group and three samples in Normal-Silibinin group.

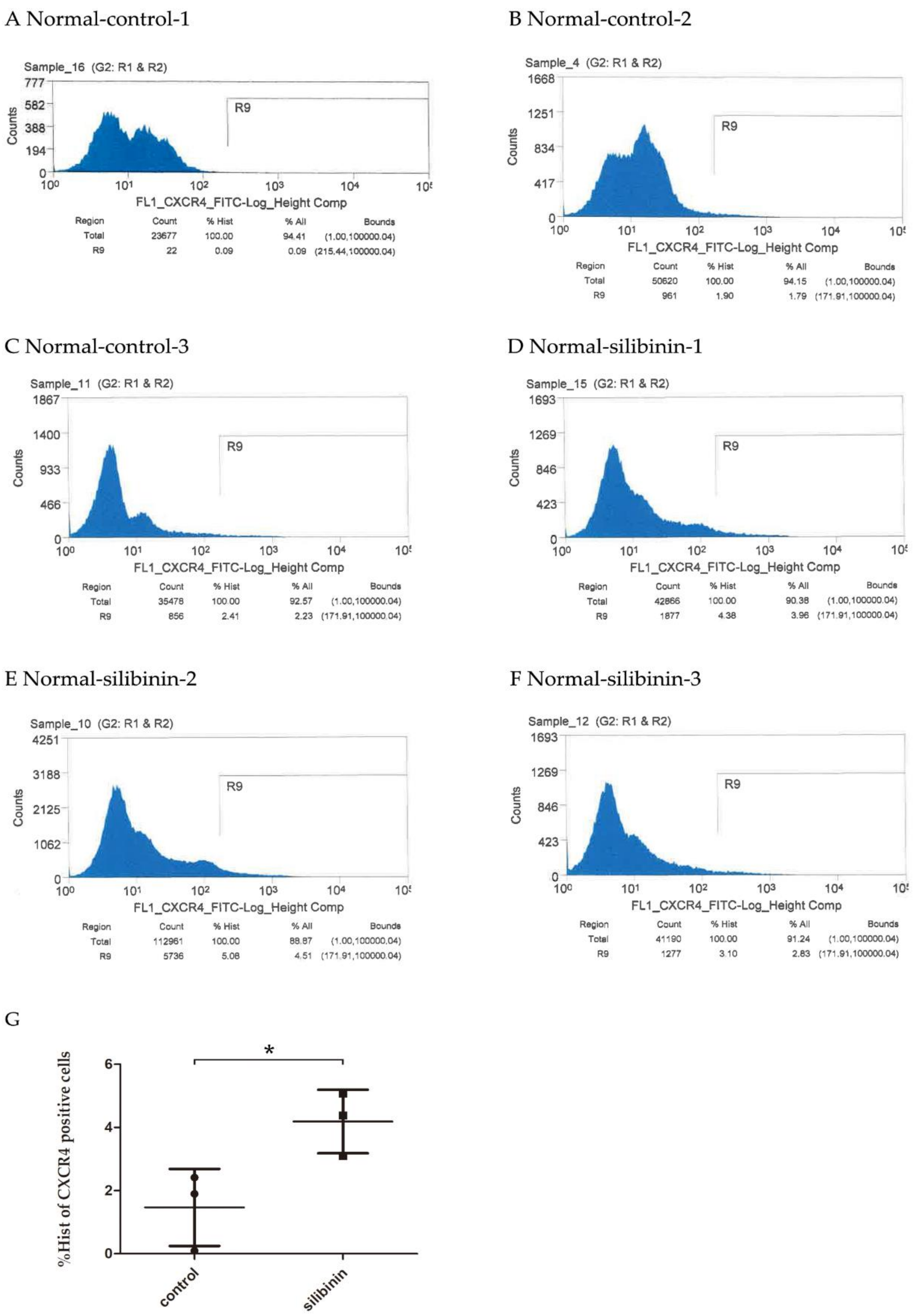

Figure S3. FCM evaluation of CXCR4 in normal rats using Moflo. (**A**–**C**) Three samples in Normal-control group, (**D**–**F**) Three samples in Normal-silibinin group. (**G**) In the BM of normal rats, silibinin upregulated the percentage of CXCR4-positive cells. *$p < 0.05$.

Table S1. Primers used for real-time PCR.

Gene	Forward primer	Reverse primer
CXCR4	5′-GCTGAGGAGCATGACAGACA-3′	5′-GATGAAGGCCAGGATGAGAA-3′
SDF-1	5′-TTTGGTCAGAGGAAGAGGGG-3′	5′-GTTTCACTACGGGCCACATC-3′
SCF	5′-TCGTGGCATGTATGGAAGAA-3′	5′-TCAGATGCCACCATGAAGTC-3′
c-Kit	5′-GATCTGCTCTGCGTCCTGTT-3′	5′-AGATGGCTGAGAAGTCCCTGT-3′
IL-6	5′-CCGGAGAGGAGACTTCACAG-3′	5′-ACAGTGCATCATCGCTGTTC-3′
TNFα	5′-TGACCCCCATTACTCTGACC-3′	5′-GGCCACTACTTCAGCGTCTC-3′
CD44	5′-TGGATCCGAATTAGCTGGAC-3′	5′-GCTTCTTCTTCTGCCCACAC-3′
CD29	5′-AACTGCACCAGCCCATTTAG-3′	5′-CCACCTTCTGGAGAATCCAA-3′
CD34	5′-GTTGGAGTCCCACAGG-3′	5′-ATTGGCCTTTCCCTGA -3′
CD10	5′-ATATGCTTGTGGAGGCTGGT-3′	5′-TTGCCCACCTCTGCTATCAA-3′
CD14	5′-CAACTTCTCAGATCCGCAGC-3′	5′-ACGCAGGGTTCCGAATAGAA-3′
ADGRE1	5′-ACACCCTTGGGAGCTACTTC-3′	5′-AGCTGCAGTTGTAGGAACCT-3′
ß-actin	5′-CTAAGGCCAACCGTGAAAAG-3′	5′-GCCTGGATGGCTACGTACA-3′

CXCR4: C-X-C chemokine receptor type 4; SDF-1: stromal cell derived factor-1; SCF: stem cell factor; IL-6: interleukin-6; TNFα: Tumor Necrosis Factor α; ADGRE1: Adhesion G protein-coupled receptor E1.
